# Paternal and Maternal Speech at 3 Months Postpartum: An Exploratory Study on the Effect of Parental Role and Birth Weight

**DOI:** 10.3390/bs14111007

**Published:** 2024-10-30

**Authors:** Erica Neri, Alessandra Provera, Francesca Agostini

**Affiliations:** Department of Psychology, University of Bologna, 40126 Bologna, Italy; erica.neri4@unibo.it

**Keywords:** infant-directed speech, mothers, fathers, preterm birth

## Abstract

Recent research highlights a growing interest in early interactions between fathers and their infants, acknowledging the significant influence these interactions have on developmental outcomes. However, there is a limited understanding of the specific characteristics of paternal infant-directed speech (IDS), especially in the context of premature birth. This study aimed to analyze the functional and morpho-syntactic features of paternal IDS to full-term (FT) and preterm (PT) infants at 3 months, comparing it with maternal communicative style. Additionally, the study explored the influence of the severity of preterm birth according to birth weight, further distinguishing between extremely low-birth-weight (ELBW) and very low-birth-weight (VLBW) infants. Seventy-one father–infant and mother–infant dyads (24 FT, 22 ELBW, 25 VLBW) were recruited at 3 months (corrected age for PTs). Parent–infant interactions were video recorded to assess lexical, syntactic, and functional aspects of paternal and maternal speech. Results revealed lower verbosity and lexical variability in paternal IDS compared to the maternal one. No differences were found between parents of the PT and FT groups. Overall, these findings could contribute to better understanding the patterns of parent–infant communications in both FT and PT dyads, confirming the importance of involving both mothers and fathers from the early stages of development.

## 1. Introduction

The quality of early parent–infant interactions is widely recognized as fundamental for the co-construction of the relationship among the baby and his/her parents, scaffolding the child’s development and promoting the areas of self-regulation, socialization, and cognitive and emotional functioning [1,2,3].

While most of the literature focused on parental interactive patterns such as sensitivity and responsivity, since the 1960s, increasing literature has studied the characteristics of the linguistic input which adults use to talk with infants and young children. This speech style, widely known as infant-directed speech [4,5], differs from adult-directed speech in various linguistic and suprasegmental adaptations, including a simplified lexicon and syntax, increased use of repetition, exaggerated high-pitched prosody, longer pauses, and slower speech rate [4,6,7,8,9,10]. Interestingly, studies analyzing patterns of maternal IDS across different cultures have found similar characteristics, particularly in the acoustic and prosodic aspects of this speech register. Cross-linguistic research, in particular, has highlighted the presence of certain universal features in IDS that serve important functions and contribute to its distinctive emotional and attention-getter qualities [11,12,13].

Research examining IDS has underscored a fundamental role in supporting infant development among several domains, such as the social, emotional, and linguistic ones. More specifically, it has been repetitively found that IDS covers a key function in facilitating the onset of social interactions, conveying of affective contents, capturing and maintaining an infant’s attention, and fostering his/her linguistic development [4,9,14,15,16]. In this regard, one significant aspect of IDS is represented by its functional features, which provide fundamental information into the purpose and pragmatics of speech. Research on parental speech has identified two main functional categories of IDS that reflect the communicative intent of the speaker, namely affect-salient and information-salient speech [17,18,19]. Affect-salient speech is primarily used to convey affective contents from the parent to the infant, thereby facilitating emotional and interactive exchanges. On the other hand, information-salient speech, which includes questions, descriptions, and directives, is used to convey information about the infant, the dyad, and the surrounding environment. Like other features of IDS, these functional aspects dynamically change over time according to the infant’s age and developmental stage.

These characteristics undergo dynamic changes over time, progressively becoming more complex as the infant grows up adapting to and supporting his/her emerging skills [10,15,19,20,21]. Specifically, during the first months postpartum, IDS is typically more affectively connotated and characterized by a higher number of questions, whereas the proportion of information-salient speech, such as descriptives and directives, increases as the infant grows up, acquires new developmental skills, and becomes able to explore the surrounding environment.

To date, the literature on IDS during the first year postpartum has predominantly focused on mother–infant dyads, with relatively less attention given to the role of paternal speech. Research involving fathers has not only been less numerous but also more discontinuous over time. The first studies which compared maternal and paternal IDS to preverbal infants have highlighted overall similar speech features. For example, Papousek et al. [22] investigated maternal and paternal speech addressed to 3-month-old infants reporting similar speech registers characterized by a simplified syntactic structure, redundancy, and slower speech tempo. Similar results were also found by Kruper and Uzgiris [23], who reported similarities in the frequent use of repetitions and questions in both maternal and paternal IDS addressed to 3- and 9-month-old infants.

Conversely, more recent studies reported little differences between the two speech styles, which regard mainly the amount of speech addressed to the infant (for a narrative review, see [24]). For example, a study by Johnson et al. [25] which investigates verbal interactive patterns among mother–infant and father–infant dyads from birth through the first 7 months postpartum, reported a higher amount of maternal speech compared to paternal during dyadic interactions. Moreover, authors found that mothers showed a higher responsiveness to an infant’s vocalizations compared to fathers, and infants tended to respond more frequently to maternal speech compared to paternal speech.

Another study by Kokkinaki [26] systematically compared different aspects of maternal and paternal IDS produced in natural interactions with their infants from 2 to 6 months after birth. Significant differences between mothers and fathers were found in terms of structural variations, quantitative differences, and similarities. Specifically, the paternal IDS structure was described as shortened and characterized by a higher rate of productions, revealing a more active and reactive speech style. Considering the amount of IDS, mothers were found to be more talkative during dyadic interactions and their speech was described as more infant- and dyad-focused compared to paternal speech. Conversely, similarities were found regarding the content of maternal and paternal IDS, such as the production of open- and close-ended questions, the use of “we” as an expression of sharing between the caregiver and the infant (emotions, physiological states, etc.), and the capability to respond to infant’s emotional expressions.

Apart from the evidence provided by these studies conducted on full-term infant populations, there is still a gap in the literature regarding the differences and similarities in maternal and paternal IDS in cases of high-risk conditions related to infant birth.

Among these, preterm birth, which means that the childbirth is anticipated before the 37th week of gestation [27], represents a complex risk factor not only for the infant [28,29,30] but also for parents, potentially affecting the establishment of positive early dyadic interactions. This risk is even more accentuated in the case of lower gestational age [31] or when the infant is born with a very low birth weight (VLBW; BW < 1500 g) or extremely low birth weight (ELBW; BW < 1000 g) [30]. According to this latter criterion, recent studies highlighted the importance of examining prematurity by specifically differentiating VLBW and ELBW populations, as they could lead to different infant developmental outcomes and also differently impact parental mental health and early dyadic interactions. For example, studies have reported a higher incidence of maternal depression in the case of an ELBW condition, as well as atypical parent–infant interaction patterns [31,32,33,34].

The preterm condition poses significant risks to an infant’s survival and health and can lead to vulnerabilities in various developmental domains, including linguistic, motor, and cognitive areas [35,36,37]. Consequently, preterm infants’ parents could face higher challenges in the transition to parenthood and are at major risk of developing negative psychological reactions (i.e., depression, anxiety, parental distress) in the postpartum period, which also constitutes an important risk factor for the onset of interactions and the relation with the infant [38,39,40,41,42].

Considering the effects of prematurity on early dyadic interactions, the presence of atypical interactive patterns seems to be related to both infant and parental variables. On one hand, due to their neuropsychological immaturity, preterm (PT) infants are described as more passive, less attentive and involved, and are more difficult during early exchanges [43,44], making it more difficult to create positive interactions. On the other hand, preterm infants’ mothers have been described as more stimulating and active, as well as less synchronized compared to full-term (FT) ones [31]. The few studies which investigated father–infant interactions in the context of prematurity reported overall similar levels of sensitivity among mothers and fathers. Interestingly, when non-sensitive behaviors were found, they tended to be more controlling for mothers, whereas fathers were described as more verbally unresponsive [45].

In the context of prematurity, studies on verbal interactive patterns focused mainly on maternal speech, reporting overall few differences in lexical and syntactic, as well as functional IDS features, at 3 and 6 months postpartum [46,47,48]. However, when considering different levels of PT severity, mothers of more severe PT infants (i.e., ELBW ones) seem to adopt a more demanding, directive, and less affective speech style compared to FT mothers and the ones of VLBW preterm infants at 3 months [33].

To the best of our knowledge, only one study so far investigated IDS among fathers and mothers addressed to PT and FT infants. Specifically, Kiepura and colleagues [49] compared vocal behaviors among mothers and fathers of PT and FT infants at 3 months, analyzing frequencies and durations of vocalizations and pauses. Results showed that maternal vocalizations were more frequent and longer compared to paternal ones, regardless of birth condition.

Given these premises and considering the lack of studies which analyzed paternal IDS patterns addressed to PT infants, the aim of our study was to contribute to fulfill this gap in the literature, describing linguistic and pragmatic features of IDS at 3 months postpartum (corrected age for PT infants). The main aim of the study was to investigate the characteristics of the speech style related to parental role by the comparison of paternal and maternal IDS in terms of syntactic, lexical, and functional features. Given the scarcity of studies on the differences between maternal and paternal IDS and considering the sometimes contradictory results, two possible outcomes can be hypothesized, one confirming and one disconfirming the differences found in the literature, which would provide greater consistency to previous studies. The second aim was to compare the characteristics of IDS directed towards preterm and full-term infants, taking into account the potential influence of PT birth weight (VLBW vs. ELBW). Concerning this topic and based on a previous study on maternal IDS directed to FT, VLBW, and ELBW infants [33], we hypothesized to find a more demanding and less affective linguistic input directed towards more severe PT infants (ELBW ones). Lastly, the effect of the interaction between parental role and birth weight was explored.

## 2. Materials and Methods

### 2.1. Study Design

The present exploratory study has been developed in the context of a broader research and follow-up project which aims to investigate the impact of premature birth on infant development, maternal and paternal mental health, and the quality of caregiver–infant interactions during the first year postpartum.

### 2.2. Participants

For this study, a total of 142 parents (71 mothers and 71 fathers) were recruited. Of these, 25 couples were parents of infants born full-term (FT) after the 36th week of gestation and with a birth weight > 2500 g. Families of FT infants were recruited for the study through a network established in collaboration with antenatal classes provided by the Health Services of Cesena. This network aimed to facilitate the recruitment of pregnant mothers for research projects and follow-up studies promoted by the Developmental Psychodynamic Laboratory of the Department of Psychology (University of Bologna), as already conducted for previous publications [33,34]. The remaining 46 couples were parents of PT infants, born before the 32nd week of gestation and with a birth weight < 1500 g, who had been hospitalized in the neonatal intensive care unit (NICU) of Bufalini Hospital (Cesena, Italy). The PT group was then divided into two subgroups according to the infants’ birth weight; 24 couples were included in the VLBW group (birth weight between 1000 and 1500 g) and 22 couples in the ELBW group (birth weight < 1000 g).

Exclusion criteria were the same for all the participants and included the presence of infant neurological disorders, genetic syndromes or other medical conditions or complications, the presence of previous or present psychiatric conditions in mothers and/or fathers, and a lack of fluency in the Italian language.

The research project was approved by the Ethical Committee of the Department of Psychology of the University of Bologna (protocol number: 0001092/2023).

### 2.3. Procedure

The assessment and data collection were conducted at 3 months postpartum (for PT infants, corrected age was considered) at the Developmental Psychodynamic Laboratory (Department of Psychology, University of Bologna, Cesena). At the beginning of the follow-up visit and assessment, which usually lasted 45 min, a psychologist asked all parents to read and sign the written informed consent. Parents were then asked to complete a short battery of questionnaires, which included a sociodemographic form and two specific and validated questionnaires—the Edinburgh Postnatal Depression Scale [50] and the Parenting Stress Index—Short Form [51]—on levels of maternal and paternal postpartum depression and parental distress in order to control the homogeneity of the groups. Once the questionnaires had been completed, which typically took five to ten minutes, a trained psychologist assessed the level of the infant’s mental and psychomotor development by administering the Griffiths Mental Development Scales—Revised version [52]. The assessment took place in a room equipped for child evaluation and video recording in the presence of the parents. After this first part of the assessment, both mothers and fathers were asked to individually participate in a 5 min session of free interaction with their infant. All the dyads were observed in the same standardized setting, which included the presence of age-appropriate toys and puppets for the infants and each free interactive session was video recorded. All materials, including administered questionnaires and video recording materials, were stored using a unique alphanumeric code in order to pseudonymize personal data. After the assessment, maternal and paternal speech directed to the infant were verbatim transcribed by a blinded trained psychologist and researcher following the Codes for the Human Analysis of Transcripts (CHAT) format [53].

### 2.4. Measures and Materials

#### 2.4.1. Sociodemographic Data

The sociodemographic characteristics of the sample were examined using an ad hoc questionnaire, which included information about parental age and education level, marital status, employment condition, and number of children. Perinatal data related to the type of delivery, the gestational period, and the infant’s characteristics at birth were also collected.

#### 2.4.2. Maternal and Paternal Postnatal Depressive Symptomatology and Parenting Stress

Mothers’ and fathers’ levels of depressive symptoms were investigated using the Edinburgh Postnatal Depression Scale [50] questionnaire, the most widely used self-report tool for the screening of postnatal depressive symptomatology in both women and men [54]. It consists of 10 items assessing the presence of perinatal depressive symptoms over the past 7 days. Each item is scored from 0 to 3 points, and the total EPDS score ranges from 0 to 30. Higher scores indicate higher levels of depressive symptoms. A validated Italian version of the EPDS questionnaire is available for both mothers [55] and fathers [56].

To investigate symptoms of parenting-related stress, all the participants were asked to complete the Italian validated version [57] of the Parenting Stress Index—Short Form (PSI-SF; [51]). This self-report tool is a 36-item questionnaire scored on a 5-point Likert scale. It generates a total score and 3 subscale scores which reflect three different dimensions, namely parental distress (PD), parent–child dysfunctional interaction (PCDI), and the perception of a difficult child (DC). Subscale scores range from 12 to 60, while the total score, which sums the subscale scores, ranges from 36 to 180, with higher scores indicating greater parenting stress levels.

#### 2.4.3. Infant Mental and Psychomotor Development

Levels of infant mental and psychomotor development at 3 months were evaluated using the Griffiths Mental Development Scales—Revised version (GMDS-R for 0–2 years; [52]). These scales have been designed for assessing infants and children from birth to 2 years. The GMDS-R provide a comprehensive evaluation through a series of developmental tests, measuring overall development across five specific domains, namely locomotor skills, personal and social development, hearing and language abilities, eye–hand coordination, and general performance. Scores are standardized, with an expected value of 100 and a standard deviation of 12. For preterm infants, the corrected age was used for scoring during assessments. The Griffiths scales are extensively used in both clinical and research settings to detect developmental delays or deficits and to track developmental progress in high-risk situations such as prematurity [31,32,45,58]. All assessments were conducted by psychologists trained in the GMDS-R and unaware of the infants’ birth weight.

#### 2.4.4. Maternal and Paternal Infant-Directed Speech

The total amount of maternal and paternal speech directed to the infant was analyzed by counting the number of utterances made during the free interaction session. An utterance, defined as any sequence of speech separated from the next by a pause longer than 1 s, served as the unit of analysis.

The lexical and syntactic features of parental IDS were examined through the following measures:

- Word tokens: the totality of words produced;
- Word types: the totality of different words produced;
- Mean length of utterance (MLU): the average number of words per utterance, which provides an index of syntactic complexity.

All these variables were analyzed according to the CHILDES system using CLAN software (version for Windows) [59], a specialized tool for examining a caregiver’s and infant speech, which allows for the verbatim transcription of verbal interactions following specific guidelines and the analysis of various linguistic features using specific command prompts.

The functional characteristics of maternal speech were analyzed using a specific coding scheme previously utilized in studies on maternal speech [33,34,60,61]. Each maternal utterance was classified into one of the following exclusive functional categories:

- Affect-salient speech: utterances aimed at sharing affective contents and maintaining the conversation (e.g., greetings, encouragement, use of onomatopoeias, singing);
- Information-salient speech: utterances intended to convey content, including giving or asking for information. This category was further divided into four exclusive subcategories: questions, labeling, descriptions, and directives;
- Attention-getters: utterances intended to attract the infant’s attention (e.g., calling the infant’s name);
- Other: incomplete or unintelligible utterances or speech not directed at the infant.

The proportion of maternal and paternal speech in the total number of utterances produced during the interaction was considered for each category and subcategory.

### 2.5. Data Analysis

Primarily, the homogeneity of the three groups in both sociodemographic characteristics (maternal and paternal age, education level, marital status, employment condition, parity, infant’s corrected age) was evaluated. Perinatal data related to the type of delivery, number of gestational weeks, the infant’s weight at birth, and the GMDS-R total score were also compared among groups. These analyses were run by a series of ANOVAs for continuous dependent variables and Pearson’s chi-square tests for categorical dependent variables. Furthermore, in order to assess the homogeneity between groups in parental clinical variables, specifically parental depression and the level of parenting stress, a series of ANOVAs were run, setting the EPDS total score and PSI total scores as dependent variables and birth group and parental role as independent ones.

In order to address the aims of the study, linguistic and functional features of IDS were compared according to the parental role, the three birth groups defined on the basis of birth weight, and the interaction between parental role and birth groups. Specifically, two multivariate analyses of covariance (MANCOVAs) were run. In the first MANCOVA, both parental role (mothers vs. fathers) and birth weight (FT, VLBW, and ELBW), as well as their interaction, were set as between-subject factors, whereas speech linguistic features (specifically, word types and tokens, MLU) were set as dependent variables. Due to the non-homogeneous distribution of certain variables (specifically gestational age and the developmental quotient, as shown in the following paragraph) and their potential influence on linguistic features, these variables were included as covariates in the statistical model. In the second model, functional categories of IDS (specifically, the proportion of affect-salient speech, labels, descriptives, questions, directives, and attention-getters) were set as dependent variables, whereas parental role, birth weight, and their interaction were identified as between-subject factors. In this second model, the developmental quotient was set as covariate.

Data analysis was conducted using IBM SPSS Statistics 24 software (IBM Corporation New York, NY, USA) and statistical significance was defined with a *p*-value < 0.05.

## 3. Results

Parental sociodemographic variables are displayed in Table 1. As mentioned before, a series of ANOVAs 2 × 3 (parental role × birth weight) were run to investigate homogeneity among groups. Statistically significant differences were found for parental age [F (5,136) = 3.4; *p* = 0.006] and years of education [F (5,136) = 2.95; *p* = 0.014]. Specifically, for parental age, a significant effect of gender [F (1,136) = 6.67; *p* = 0.011] and birth group [F (2,136) = 5.09; *p* = 0.007] emerged, indicating that mothers were overall younger than fathers, and FT parents were overall younger compared to parents of the VLBW group (Tukey post hoc: *p* = 0.006). A gender effect emerged [F (1,136) = 9.40; *p* = 0.003)] regarding years of education, indicating a higher level of education in mothers compared to fathers independently from birth groups. No significant differences among parents and groups were found in marital status, working condition, and parity condition.

Considering maternal and paternal levels of depressive symptoms and parental distress, no significant differences emerged in the EPDS and PSI scores among groups. The two models did not reach statistical significance (EPDS scores: F (5,136) = 1.70; *p =* 0.137; PSI scores: F (5,136) = 0.33; *p =* 0.894), suggesting a certain degree of homogeneity among parents.

Regarding the infant variables, displayed in Table 2, the three groups significantly differed according to gestational age, birth weight, hospitalization period before discharge, and the infant’s developmental quotient. Specifically, the infant’s gestational age and birth weight were higher in the FT group compared to both the LBW (Tukey post hoc: *p* = 0.010 and *p* = 0.001, respectively) and ELBW conditions (Tukey post hoc: *p* = 0.010 and *p* = 0.001, respectively) and in the VLBW group compared to the ELBW one (Tukey post hoc: *p* = 0.031 and *p* = 0.001, respectively). Similarly, FT infants showed higher developmental quotient scores compared to VLBW (Tukey post hoc: *p* = 0.028) and ELBW ones (Tukey post hoc: *p* = 0.001) and VLBW infants reported higher scores compared to ELBW ones (Tukey post hoc: *p* = 0.025).

Given the non-homogeneous distribution of some parental and infant variables, a series of correlations were first run in order to check their possible association with parental speech measures and to identify the presence of covariates.

No significant associations emerged among sociodemographic variables (parental age and years of education), while the infant developmental quotient and gestational age at birth were positively correlated with all the lexical and syntactic measures as follows: MLU (Pearson’s r = 0.168; *p* = 0.046; Pearson’s r = 0.200; *p* = 0.019, respectively), types (Pearson’s r = 0.248; *p* = 0.003; Pearson’s r = 0.171; *p* = 0.045, respectively), and tokens (Pearson’s r = 0.251; *p* = 0.003; Pearson’s r = 0.161; *p* = 0.050, respectively). Regarding the relation between functional features and socio-demographic variables, a positive correlation was found only between labels and the developmental quotient (Pearson’s r = −0.197; *p* = 0.018). Consequently, only gestational age and the developmental quotient were included as covariates in the following analyses.

### 3.1. Lexical and Syntactic Features of Maternal and Paternal IDS at 3 Months

To investigate the first research question of our study, two series of multivariate analyses of covariance (MANCOVAs) were run. The first MANCOVA included parental role and birth group as independent variables and the MLU, word types, and tokens as dependent variables. Birth weight, gestational age at birth, and the infant developmental quotient were added as covariates. Results are displayed in Table 3.

The multivariate analysis of covariance highlighted a statistically significant effect of parental role [F (3,129) = 2.72, *p* = 0.047, partial η^2^ = 0.06] and of the interaction parental role × birth weight [F (6,260) = 2.14, *p* = 0.049, partial η^2^ = 0.05] but not as a main effect of birth weight [Pillai’s trace: F (6,260) = 2.10, *p* = 0.054, partial η^2^ = 0.05].

A univariate analysis showed a statistically significant parental role effect on the indexes of word types [F (1,133) = 6.03; *p* = 0.015; partial η^2^ = 0.05] and tokens [F (1,133) = 8.24; *p* = 0.005; partial η^2^ = 0.06], which was higher in mothers compared to fathers, whereas no statistically significant effects were found for the MLU index. Despite the multivariate model for the interaction effect being statistically significant, no statistically significant effects emerged from the univariate analyses (alle *ps* > 0.05).

### 3.2. Functional Features of Maternal and Paternal IDS at 3 Months

Secondly, a MANCOVA model including parental role and birth group as independent variables and functional categories of IDS (affect-salient speech, labels, descriptives, questions, directives, attention-getters) as dependent variables was run. As preliminary correlations showed significant associations between the proportion of labels and the developmental quotient (Pearson’s r = −0.200; *p* = 0.017), this last variable was included as covariate in the analysis.

**Figure 1:**
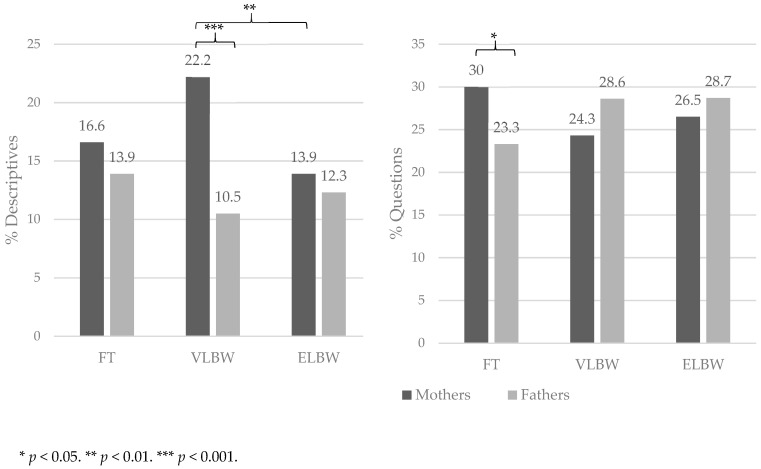
Percentages for descriptive interactions and questions related to parental roles and birth weight.

Results are displayed in Table 4. The multivariate analysis of covariance highlighted a statistically significant effect of parental role [F (6,129) = 2.73, *p* = 0.016, partial η^2^ = 0.12] and of the interaction parental role × birth group [F (12,260) = 2.12, *p* = 0.016, partial η^2^ = 0.09], while the main effect of birth group was not statistically significant [Pillai’s trace: F (12,260) = 1.21, *p* = 0.271, partial η^2^ = 0.05]. A statistically significant effect of parental role emerged for the proportion of descriptive utterances produced, which was higher in mothers compared to fathers regardless of the birth weight of the infant [F (1,135) = 12.8; *p* < 0.001; partial η^2^ = 0.10]. Two statistically significant interaction effects emerged in both the functional categories of descriptives [F (2,135) = 5.57; *p* < 0.005; partial η^2^ = 0.08] and questions [F (2,135) = 3.34; *p* = 0.038; partial η^2^ = 0.05] [Figure 1]. Specifically, mothers of VLBW infants tended to produce more descriptive utterances compared to fathers of the same group (Bonferroni post hoc: *p* < 0.001) and mothers of ELBW infants (Bonferroni post hoc: *p* = 0.002), whereas mothers of FT infants produced more questions compared to fathers of the same group (Bonferroni post hoc: *p* = 0.039).

## 4. Discussion

Early caregiver–infant interactions play a key role for the infant’s development and psychological health. One important aspect of dyadic interactions is represented by infant-directed speech, which covers several developmental and affective functions, influencing an infant’s growth in several developmental trajectories and facilitating the establishment of positive relations between the infant and the caregiver.

Given the lack of studies on IDS in the literature, the aim of this study was to contribute to enriching the literature on early father–infant interactions, exploring the characteristics of paternal language directed to the infant at the end of the first trimester postpartum in the case of full-term (FT) and preterm (PT) birth. Specifically, our purpose was to compare maternal and paternal speech in interactions with their infants, taking into consideration the possible impact of different levels of prematurity according to birth weight.

### 4.1. Lexical and Syntactic Features of Maternal and Paternal IDS at 3 Months

Considering the lexical and syntactic features of IDS, a significant main effect of parental role emerged for the number of word types and tokens produced, whereas no main effect of parental role was observed for the mean length of the utterance (MLU). Specifically, both word types and tokens, which reflect the number of different words produced and the speech amount addressed to the infant, respectively, were significantly lower in fathers compared to mothers, regardless of the birth weight of their infants. These results highlight how in our sample, paternal IDS was overall characterized by a lower lexical variability and verbosity compared to maternal IDS, suggesting that mothers tend to be more talkative than fathers during dyadic interactions. Conversely, the MLU was not different between mothers and fathers, suggesting a similar degree of syntactic complexity in maternal and paternal speech. These findings are coherent with the previous literature on parental IDS, which reported a higher amount of linguistic input produced by mothers compared to fathers in interactions with their babies, as well as similar syntactic properties of maternal and paternal speech (i.e., [25,26]).

Conversely, the main effect of birth weight did not seem to influence lexical and syntactic features of parental speech, as shown by the absence of statistically significant differences among groups in both the number of word types and tokens, as well as the MLU. This finding is in line with previous studies, which reported overall no or small differences in maternal IDS addressed to their FT and PT infants in the first semester after birth [33,47,48]. However, none of these studies explored the role of fathers and only one [33] investigated different levels of prematurity. Thus, our study confirms and extends these findings also to paternal speech, further comparing ELBW and VLBW infants.

Taken together, these results seem to suggest a certain degree of gender specificity in lexical and syntactic registers directed to infants that is independent of birth weight, as highlighted by the absence of a significant interaction effect between parental role and birth weight.

### 4.2. Functional Features of Maternal and Paternal IDS at 3 Months

Moving on to functional features of IDS, we found a lower number of descriptive utterances in paternal speech compared to maternal speech. This result is coherent with previous studies. Specifically, a study by Kokkinaki [26], which investigated parental IDS from 2 to 6 months after birth, reported a higher number of declarative utterances in mothers compared to fathers. Furthermore, another study by Venuti and colleagues [60] found the same results in mothers and fathers of older children (2 to 4 years old) of typical and atypical development. We might hypothesize that this pattern could reflect a higher predisposition in mothers to describe to their infants the context in which the interaction is taking part. This tendency could reflect an aspect of parenting which is already present from the early stages of development and that transcends the infant’s birth weight, as suggested by the absence of a significant main effect of birth weight. Interestingly, the significant interaction effect in which mothers of ELBW infants produced less descriptives compared to VLBW ones could reflect an adaptation of their speech, which could be related to the birth condition of their infant. Indeed, severe preterm infants tend to produce less vocalizations compared to FT ones [62,63]. It could be hypothesized that the perception of a higher immaturity of their infants, and probably also their greater passivity during interactions, could lead mothers of more severe PT infants to recall more frequently their attention, rather than describing the surrounding environment, with the aim of eliciting a response. This hypothesis is somehow supported by the higher, despite not significant, proportion of attention-getters produced by mothers of ELBW infants compared to VLBW ones.

A further significant effect emerged for the proportion of questions. In general, questions are a prominent type of utterance in both maternal and paternal IDS, especially in the first months after birth. Consistent with the existing literature, our sample showed also that questions were the second most common functional category used by parents, following affect-salient speech. In the present study, we found a significant difference only in the comparison between FT mothers and FT fathers, where the first showed a higher number of questions than the latter. Since previous studies, different to our findings, have found a similar number of questions produced by the fathers and mothers of FT infants [26], our findings need further investigation.

Considering all the other functional features of IDS, our results revealed similar patterns in all dyads. In fact, both the proportion of affective contents, labels, directives, and attention-getter utterances did not significantly differ either according to parental role, birth weight, or their interaction. This homogeneous trend in the functional contents of IDS produced by mothers and fathers aligns with previous findings in the literature, which reported overall similar speech styles in both parents of FT infants (see, for example, [26]), extending these results also to the case of prematurity.

As previously mentioned, one of the most important aspects of IDS during the first phases of development is its affective connotation, which is crucial to convey and share affective contents with the infant, establish positive interactions, and build secure attachment bonding [1,2]. In our sample, almost half of the speech input produced by both mothers and fathers of the three groups was affect-salient speech, suggesting a similar tendency of both mothers and fathers to engage their infant in sensitive and attuned interactions, regardless of the infants’ status. Conversely, the proportion of directive utterances was small and similar among parents of all the groups, suggesting the presence of low levels of intrusiveness in the speech style of both mothers and fathers, independent of the birth condition. Furthermore, the use of labels, which represents a subcategory of information-salient speech, usually becomes more frequent starting from the second year as the infant grows up and starts exploring and orienting his/her attention to the surrounding environment (for example, pointing or looking at objects). In this sense, labeling objects becomes functional to scaffold the infant’s linguistic and vocabulary acquisition later in development, whereas during the first half of the first year, this category is usually less salient for the infant and less functional for the development of early relations [19]. The absence of differences among parents and groups in the proportion of this functional category could suggest a certain capability to adapt the contents of speech according to the infant’s age and level of development, which would express a sensitive approach towards the infant’s needs and capacities.

Taken together, the present study could suggest an overall adequate quality of the vocal interaction of all parents with their infants, regardless of parental role and birth weight.

In general, our study seemed to align with previous findings in the literature on maternal and paternal IDS, thus extending the knowledge of gender-related differences and similarities, also in the PT population. Despite preliminary findings, these results should encourage the continuation of orienting research to fathers and the importance of their involvement in an infant’s caregiving, as they play an important protective role to support early dyadic interactions.

Conversely, the absence of a significant effect of preterm status, especially in cases of severe prematurity, is not consistent with the literature on maternal interactive behaviors, and IDS at 3 months after the birth reported a significant effect of more severe premature birth according to birth weight. Specifically, mothers of ELBW infants seemed to interact with their infants by displaying more intrusive behaviors [31,32] and less prototypical IDS patterns [33,34]. However, when studies on interactive patterns considered both infant birth weight and parental role, other researchers did not find a significant effect of birth status on early interactive behaviors [45,64,65], suggesting that the effect of prematurity could be less significant when parent gender is also considered. This finding underscores the importance of evaluating the parental dyad rather than focusing solely on the individual contributions of the mother or father.

### 4.3. Limitations and Future Directions

Some limitations should be discussed. First, the small sample size of our research makes it difficult to generalize our results to the FT and PT populations. Even if recruiting parental dyads of both mothers and fathers could be challenging, further studies should enlarge the number of participants involved in order to possibly confirm the results of this study. Furthermore, our study is constrained by its focus on a single age point. Longitudinal studies are needed to explore the developmental trajectories of maternal and paternal IDS patterns directed to PT and FT infants.

Secondly, we controlled homogeneity for parental symptomatology among groups, but a specific effect on our dependent variables was not investigated despite previous studies reporting significant effects of maternal symptomatology on mothers’ IDS [33,34]. Related to this issue, the use of self-reporting could be not entirely suitable for identifying gender-related differences in the manifestation of postnatal depressive symptoms [66], especially for fathers.

Another important limitation in the generalization of our results is that they refer only to the Italian linguistic and cultural context. However, since the cultural context of origin may influence how parental roles are expressed and, consequently, affect maternal and paternal interactive behaviors, it would be desirable in the future to conduct cross-cultural studies to explore the presence of similarities and differences related to both native language and cultural background. With regard to this last point, we would also highlight that the sole representation of the mother–father couple is not sufficient to describe the plurality of parental roles that exist in modern society. Today, there is an increasing variety of family structures, including, among others, single-parent and same-sex parent families. Although our study focused on mother–father parental couples, we believe it is becoming more important to extend research to these other family forms in order to foster a deeper understanding of both the specificities and similarities that characterize the plurality of parenting experiences.

## 5. Conclusions

This study aimed to provide a contribution to deepening the knowledge of the differences and similarities between maternal and paternal speech both in the case of FT birth and PT birth.

As previously discussed, results revealed overall small differences between mothers and fathers and among groups of PT and FT infants, as well as in the case of more severe premature birth. Even if promising, we consider that our findings could be partially explained by considering the characteristics of data collection and recruitment of this research. In the case of PT groups, parents were both involved in a research and follow-up project which supports families of PT infants from birth to 24 months of corrected age, following and monitoring the infant’s development as well as parental well-being. We might hypothesize that the parents of preterm infants who agreed to participate in the study are inherently driven by a strong motivation, which could in turn reflect greater parental sensitivity and a higher attentiveness to the developmental needs of their children. All these aspects could constitute protective factors for the establishment of positive dyadic interactions, which in fact were, in our sample, similar to the ones found in FT dyads.

Besides these considerations, we hope that this study may contribute to underlining two main aspects. The first one is the importance of including fathers in assessment programs and interventions, as they play a fundamental role in supporting an infant’s development and psychological health. To date, the literature on IDS has mainly focused on maternal input, because mothers have historically been considered as “primary caregivers”, whereas fathers have been for a long time considered as less involved in infant caretaking and thus less crucial in affecting an infant’s development. Consequently, the idea that mothers are more involved in an infant’s caring and fathers in working duties has been predominant, leading fathers to become less involved also in follow-up and research appointments both for working reasons and cultural ones [24,67]. Luckily, the spread of a more egalitarian perception of parental roles as well as scientific evidence of similar levels of sensitivity among mothers and fathers [68] has contributed to orient the literature on the paternal role and its importance for an infant’s development and well-being.

The second aspect is related to the importance of raising awareness and providing adequate training for hospital staff to foster an environment that supports early mother–infant and father–infant interactions. It is particularly crucial to recognize the significant role of maternal and paternal vocal input during the first period postpartum in order to develop programs of early interventions oriented in sustaining parenting roles and the development of a positive caregiver–infant bonding.

## Figures and Tables

**Table 1:** Parents’ sociodemographic and clinical characteristics and differences among groups.

	Mothers (N = 71)			Fathers (N = 71)			F (5,136)	*p*
	FT (N = 24)	VLBW (N = 25)	ELBW (N = 22)	FT (N = 24)	VLBW (N = 25)	ELBW (N = 22)		
Age ^a^	33.4 (5.05)	36.8 (6.18)	36.1 (5.02)	36 (5.85)	39.4 (6.01)	38 (4.33)	3.41	0.006 **
Years of education ^a^	14.9 (2.84)	15.1 (2.04)	14.1 (2.95)	12.4 (2.99)	13.6 (2.04)	13.8 (2.84)	2.95	0.014 *
Working condition ^b^							6.31	0.177
Employed	23 (96)	25 (100)	21 (95)	24 (100)	25 (0)	21 (95)		
Unemployed	1 (4)	0 (0)	1 (5)	0 (0)	0 (0)	1 (5)		
Marital status ^b^							1.99	0.370
Married/cohabit	23 (96)	25 (100)	22 (100)	23 (96)	25 (100)	22 (100)		
Other	1 (4)	0 (0)	0 (0)	1 (4)	0 (0)	0 (0)		
Parity ^b^							3.85	0.146
Nulliparous	23 (96)	19 (76)	18 (82)	23 (96)	19 (76)	18 (82)		
Multiparous	1 (4)	6 (24)	4 (18)	1 (4)	6 (24)	4 (18)		
EPDS score ^a^	4.83 (3.14)	6.76 (3.52)	5.82 (5.17)	4.58 (2.69)	4.44 (3.07)	4.32 (3.88)	1.70	0.137
PSI total score ^a^	59.9 (13.3)	59.3 (13.5)	61.3 (15.6)	56.3 (11.2)	57.9 (13.9)	59.1 (18.8)	0.33	0.894

^a^ Interval data are expressed as means (with standard deviations in parentheses). ^b^ Categorical data are expressed as frequencies (and % in parentheses). * *p* < 0.05. ** *p* < 0.01.

**Table 2:** Infants’ characteristics and differences among groups.

	FT (N = 24)	VLBW (N = 25)	ELBW (N = 22)	F (2,68)	*p*
Infant’s mean age (in months) ^a^	2.97 (0.21)	3.07 (0.19)	3.06 (0.19)	1.98	0.145
Gestational age at birth (in weeks)	37.4 (5.50)	29.7 (1.95)	27 (1.74)	52.5	<0.001 ***
Birth weight (in grams)	3607 (422)	1301 (136)	870 (153)	659	<0.001 ***
GMDS-R total score ^a^	114 (7.96)	109 (7.07)	103 (7.58)	12.2	<0.001 ***

Data are expressed as means (with standard deviations in parentheses). ^a^ Calculated based on corrected age for preterm infants. *** *p* < 0.001.

**Table 3:** Means, standard deviations, and univariate analyses of syntactic and lexical features of IDS.

	Birth Weight			Parental Role		FT		VLBW		ELBW		Birth Weight			Parental Role			BW × PR		
	FT (N = 48)	VLBW (N = 50)	ELBW (N = 44)	Fathers (N = 71)	Mothers (N = 71)	Fathers (N = 24)	Mothers (N = 24)	Fathers (N = 25)	Mothers (N = 25)	Fathers (N = 22)	Mothers (N = 22)	F	*p*	Part η^2^	F	*p*	Part η^2^	F	*p*	Part η^2^
Word tokens	221 (43.1)	197 (17.1)	172 (26.9)	179 (8.93)	215 (8.93)	189 (62.5)	245 (49.6)	198 (87.2)	201 (88.5)	151 (67.4)	191 (69.1)	0.85	0.426	0.01	8.24	0.005 **	0.06	2.01	0.128	0.03
Word types	75.9 (15.6)	73.0 (6.20)	73.6 (9.63)	68.7 (3.22)	79.8 (3.22)	67.5 (16.1)	84.4 (16.1)	70.5 (7.23)	75.6 (7.23)	68.1 (10.4)	79.2 (10.4)	0.02	0.976	0.01	6.03	0.015 *	0.05	0.57	0.565	0.01
MLU	2.33 (0.39)	3.34 (0.15)	3.41 (0.24)	2.93 (0.08)	3.12 (0.08)	2.21 (0.40)	2.45 (0.40)	2.89 (0.18)	3.28 (0.18)	3.45 (0.26)	3.37 (0.26)	2.03	0.135	0.03	2.68	0.104	0.02	1.54	0.217	0.02

Data are expressed as estimated marginal means (with standard errors in parentheses) for interval data. * *p* < 0.05. ** *p* < 0.01.

**Table 4:** Means, standard deviations, and univariate analyses of functional features of IDS.

	Birth Weight			Parental Role		FT		VLBW		ELBW		Birth Weight			Parental Role			BW × PR		
	FT (N = 48)	VLBW (N = 50)	ELBW (N = 44)	Fathers (N = 71)	Mothers (N = 71)	Fathers (N = 24)	Mothers (N = 24)	Fathers (N = 25)	Mothers (N = 25)	Fathers (N = 22)	Mothers (N = 22)	F	*p*	Part η^2^	F	*p*	Part η^2^	F	*p*	Part η^2^
Affect-salient speech	48.2 (2.61)	44.36 (2.35)	45.3 (2.73)	47.95 (1.97)	44.2 (1.97)	50.5 (3.54)	46 (3.54)	45.7 (3.33)	43 (3.33)	47.6 (3.7)	43.6 (3.71)	0.62	0.536	0.01	1.76	0.186	0.03	0.04	0.961	0.01
Labels	1.07 (0.48)	2.08 (0.43)	1.50 (0.50)	1.57 (0.36)	1.53 (0.36)	1.08 (0.66)	1.07 (0.66)	2.30 (0.62)	1.86 (0.62)	1.34 (0.68)	1.65 (0.68)	1.26	0.287	0.02	0.01	0.926	0.00	0.17	0.843	0.01
Descriptives	15.3 (1.36)	16.5 (1.23)	12.8 (1.42)	12.28 (1.03)	17.50 (1.03)	13.92 (1.85)	16.61 (1.85)	10.5 (1.74)	22.5 (1.74)	12.3 (1.94)	13.9 (1.94)	1.86	0.159	0.03	12.8	<0.001 ***	0.10	5.57	0.005 **	0.08
Directives	3.10 (1.03)	4.86 (0.93)	3.96 (1.08)	4.26 (0.78)	3.68 (0.78)	4.72 (1.40)	1.48 (1.40)	5.47 (1.32)	4.24 (1.32)	2.6 (1.46)	5.33 (1.46)	0.86	0.444	0.01	0.27	0.603	0.01	2.42	0.093	0.04
Questions	26.7 (1.73)	26.4 (1.56)	27.6 (1.81)	26.91 (1.31)	26.95 (1.31)	23.3 (2.35)	30.0 (2.35)	28.6 (2.21)	24.3 (2.21)	28.7 (2.46)	26.5 (2.46)	0.12	0.884	0.02	0.01	0.984	0.01	3.34	0.038 *	0.05
Attention-getters	4.95 (1.22)	4.78 (1.10)	6.65 (1.28)	5.58 (0.93)	5.31 (0.93)	5.27 (1.66)	4.64 (1.66)	5.74 (1.56)	3.71 (1.56)	5.72 (1.73)	7.59 (1.73)	0.69	0.502	0.01	0.04	0.840	0.00	0.74	0.478	0.01

Data are expressed as estimated marginal means (with standard errors in parentheses) for interval data. * *p* < 0.05. ** *p* < 0.01. *** *p* < 0.001.

## Data Availability

The data presented in this study are available upon request from the corresponding author. The data are not publicly available due to privacy and ethical reasons.
